# Effects of Blood Flow Restriction Resistance Exercise Versus Traditional Resistance Exercise in Voluntary Exhaustion on Quadriceps Muscle Adaptations in Untrained Young Males: A Randomized Trial

**DOI:** 10.3390/medicina61050804

**Published:** 2025-04-26

**Authors:** Mustafa Şakir Akgül, Hüseyin Şahin Uysal, Nevin Köremezli Keskin, Tuğba Çetin, Merve Başdemirci, Melike Nur Akgül, Zehra Yıldız, Ebubekir Çiftçi, Recep Soslu

**Affiliations:** 1 Department of Coaching Education, Hasan Dogan Faculty of Sport Sciences, Karabuk University, Karabuk 78000, Turkey; tugbacetin@karabuk.edu.tr (T.Ç.); yzehra793@gmail.com (Z.Y.); 2 Department of Physical Education and Sport, Faculty of Sport Sciences, Burdur Mehmet Akif Ersoy University, Burdur 15030, Turkey; hsuysal@mehmetakif.edu.tr; 3 Department of Radiology, Karabuk University Training and Research Hospital, Karabuk 78000, Turkey; nevinkoremezlikeskin@karabuk.edu.tr (N.K.K.); gokceoglumrv@gmail.com (M.B.); 4 Eskipazar Vocational School, Karabuk University, Karabuk 78000, Turkey; nurakgul@karabuk.edu.tr; 5 Department of Movement and Exercise Sciences, The Institute of Graduate Programs, Istanbul Gelişim University, İstanbul 34315, Turkey; msc.ebuciftci@gmail.com; 6 Department of Coaching Education, Faculty of Sports Sciences, Karamanoğlu Mehmetbey University, Karaman 70200, Turkey; recepsoslu@kmu.edu.tr

**Keywords:** vascular occlusion, ultrasonography, hypertrophy, muscle strength, cross-sectional area

## Abstract

*Background and Objectives:* This study compared the effects of blood flow restriction resistance exercise (BFR-RE) and high-load resistance exercise (HL-RE) in voluntary exhaustion on quadriceps muscle adaptations in untrained young males. *Materials and Methods:* This study used a randomized controlled design that included 30 untrained young males (age = 21.42 ± 2.51). The BFR-RE group performed leg extension exercises with 60% occlusion pressure and 30% of one maximum repetition in volitional exhaustion. The same exercise was conducted at 70% 1RM in the HL-RE group. Fourteen variables were used to evaluate the intervention efficacy, including muscle thickness, stiffness, strength, cross-sectional area (CSA), and subcutaneous fat thickness. Analyses were reported using frequentist and Bayesian approaches. The Bayes factor (BF10 and BFincl) was interpreted based on negative and positive values. *Results:* The results revealed that the main effect of time was statistically significant for muscle strength, thickness, CSA, and stiffness (*p* < 0.05, BFincl > 1) and, in intragroup comparisons, both groups showed improvements in these parameters (*p* < 0.05, BF10 > 1). A statistically significant decrease in subcutaneous fat thickness was observed in the BFR-RE group (*p* < 0.05, BF10 > 1), while this change was not observed in the HL-RE group (*p* > 0.05, BF10 < 1). Similarly, a statistically significant increase in right rectus femoris muscle stiffness was detected in the BFR-RE group (*p* < 0.05, BF10 > 1) but not in the HL-RE group (*p* > 0.05, BF10 < 1). Furthermore, time’s main effect was statistically insignificant for thigh circumference (*p* > 0.05, BFincl < 1). The group × time interaction was statistically significant only for peak power leg flexion left (*p* < 0.05, BFincl > 1), and a statistically significant difference in favor of the BFR-RE group was observed in the intergroup comparisons (*p* < 0.05, BF10 > 1). *Conclusions:* In conclusion, BF-RE exercise with voluntary exhaustion may be as effective as HL-RE for hypertrophic adaptations in untrained young males.

## 1. Introduction

Low-load resistance exercise with blood flow restriction (BFR-RE) offers a compelling alternative to high-load resistance exercise (HL-RE), particularly in situations where high loads are impractical due to various constraints [1]. By way of illustration, apprehensions pertaining to safety protocols, physical constraints, or persistent rehabilitative necessities may exist. Furthermore, BFR-RE yields noteworthy hypertrophic and strength enhancements with diminished external loads, a factor of paramount importance for demographic groups wherein substantial weightlifting could elevate the potential for injury, such as geriatric individuals or those with particular musculoskeletal pathologies.

Loenneke et al. reported that BFR-RE elicits comparable gains in muscular strength and hypertrophy to high-intensity training, thereby substantiating its efficacy in recruiting muscle fibers typically engaged under higher load conditions [2]. Conversely, subsequent research by Groennebaek et al. suggests a potentially divergent effect of BFR on mitochondrial protein synthesis compared with HL-RE, underscoring the necessity for a more refined comprehension of its physiological mechanisms [3]. Furthermore, it has been established that the discomfort inherent in BFR-RE can induce elevated muscle activation, in some instances approaching levels achieved through traditional high-load exercises [4].

BFR-RE involves restricting blood flow to the working muscles, inducing a state of ischemia [5]. A significant advantage of BFR-RE is its ability to stimulate muscle hypertrophy and strength adaptations using light external loads (20–30% of one-repetition maximum [1RM]), which can be comparable to the results achieved with high-load training programs that utilize 70–85% of 1RM [6]. Consequently, BFR training has gained popularity in athletic performance and rehabilitation contexts over the past few decades.

Quadriceps strength and power are essential for enhancing athletic performance and facilitating the return to unrestricted sporting activities following injury [7]. A previous review indicated that weakened quadriceps strength is a significant risk factor for symptomatic and functional decline in the knee during daily living and sports recreational activities [8]. Numerous electromyographic (EMG) studies have suggested that single- and multi-joint exercises elicit different muscle activation patterns. For instance, single-joint exercises targeting the quadriceps, such as leg extensions, demonstrate higher EMG amplitudes than multi-joint lower extremity exercises, such as leg presses and squats [9,10]. Resistance training, characterized by high mechanical tension, remains the cornerstone for promoting muscle hypertrophy [11]. Research indicates that higher training intensities are associated with greater hypertrophy up to a certain threshold [12]. Although both light and heavy loads have been reported to elicit similar muscle growth when sets are taken to failure [13,14], studies have argued that high-repetition training with light loads may result in greater central fatigue [15].

The existing literature comparing the effects of BFR-RE and HL-RE primarily focuses on the quadriceps and hamstring muscle groups, along with their associated exercises [16,17]. Most studies that compare BFR-RE and HL-RE interventions in trained individuals have predominantly employed repetition schemes consisting of sets of 15 repetitions or a fixed protocol of 75 repetitions. These studies typically utilize training programs lasting 6 to 8 weeks, conducted 2 to 3 times per week, with post-intervention assessments performed 48 to 72 h after the final training session [16,17].

However, many of these studies have been conducted on trained individuals, often employing 6-week training programs performed 2 to 3 times per week, with 3 to 4 sets per exercise and a variable number of repetitions per set. While the recent research suggests that total sets performed to voluntary exhaustion or near-voluntary exhaustion are the most valid descriptor of volume [18] and report comparable hypertrophic gains with both high-load and low-load resistance training protocols to voluntary exhaustion [13,19], the aforementioned studies did not utilize a voluntary exhaustion training protocol with BFR.

Furthermore, the studies frequently did not incorporate a minimum 4–5-day post-training recovery period recommended to mitigate the confounding effects of edema on hypertrophy assessments [20,21]. Because muscle growth following resistance training may result from the accumulation of contractile proteins and extracellular fluid (ECF) accumulation secondary to muscle damage [22], this ECF accumulation, particularly pronounced with high training volumes, can manifest as edema or inflammation, leading to a transient increase in muscle size that may obscure accurate hypertrophic signals and compromise the measurement precision. While edema and hypertrophy resulting from ECF accumulation are commonly assessed using techniques such as muscle girth measurements, ultrasonography, or magnetic resonance imaging (MRI), none of these methods definitively differentiate between edema and true hypertrophy [20].

Although the evidence supporting comparable muscle strength and hypertrophy gains with BFR-RE and HL-RE is accumulating, to our knowledge, no prior studies have employed ultrasonography to assess quadriceps adaptations one week post-intervention following an eight-week BFR-RE and HL-RE training program (three sessions per week, three sets to voluntary exhaustion) in untrained individuals.

In this context, this study aimed to compare the effects of BFR-RE and HL-RE on quadriceps muscle strength, thickness, stiffness, and cross-sectional area (CSA) in untrained males, with sets performed in voluntary exhaustion. We hypothesized that there would be no statistically significant difference in hypertrophic responses between the BFR-RE group and the HL-RE group in all parameters or that significant results would favor BFR-RE.

## 2. Materials and Methods

### 2.1. Study Design

This study performed a quasi-experimental, double-blind, and randomized controlled repeated measures design. The study protocol was pre-registered on ClinicalTrials.gov (Identifier: NCT06746792). CONSORT guidelines were adhered to in order to enhance the reporting quality of this study [23]. Details regarding the CONSORT checklist are provided in Supplementary Materials, and the study files are available for open access through the Open Science Framework (OSF) at https://osf.io/mw2hn/ (accessed on 15 March 2025).

#### 2.1.1. Experimental Design

This study’s dependent variables were one-repetition maximum strength (1RM), muscle strength, thickness, stiffness, subcutaneous fat thickness, and CSA. Two different training formats, BFR-RE and HL-RE, constituted this study’s independent variables. All exercise sessions were conducted at the fitness center of the Faculty of Sport Sciences, Karabuk University. Ultrasound (USG) data were evaluated in the Karabuk University Training and Research Hospital Radiology Department. During the experiment, the ambient temperature was kept between 21 and 23 °C, and the relative humidity was maintained at 40–50%. All training sessions were performed at the same time of day to control exercise performance and circadian changes in physiological responses. A strength conditioning specialist researcher designed the exercise protocol. An independent researcher recorded the participants’ progress in each exercise session, and two radiologists with over 5 years of experience performed muscle thickness, CSA, and stiffness measurements. Radiologists were blinded to group allocation. The statistical analyses of this study were performed by an independent researcher who was not directly involved in the experimental process.

#### 2.1.2. Participants

Thirty male undergraduate students (age: 22.13 ± 3.29 years; height: 180.93 ± 7.88 cm; body mass: 23.04 ± 3.35 kg) from the Faculty of Sports Sciences at Karabuk University who reported not engaging in regular exercise were included in this study. As an inclusion criterion, only male participants with prior experience in lower body resistance training but with no more than two years of overall resistance training experience were included in this study. Additionally, participants were required to be free of orthopedic disorders or hypertension (defined as >140/90 mm Hg) that would hinder their ability to perform lower body resistance training. All participants reported right leg dominance.

**Figure 1:**
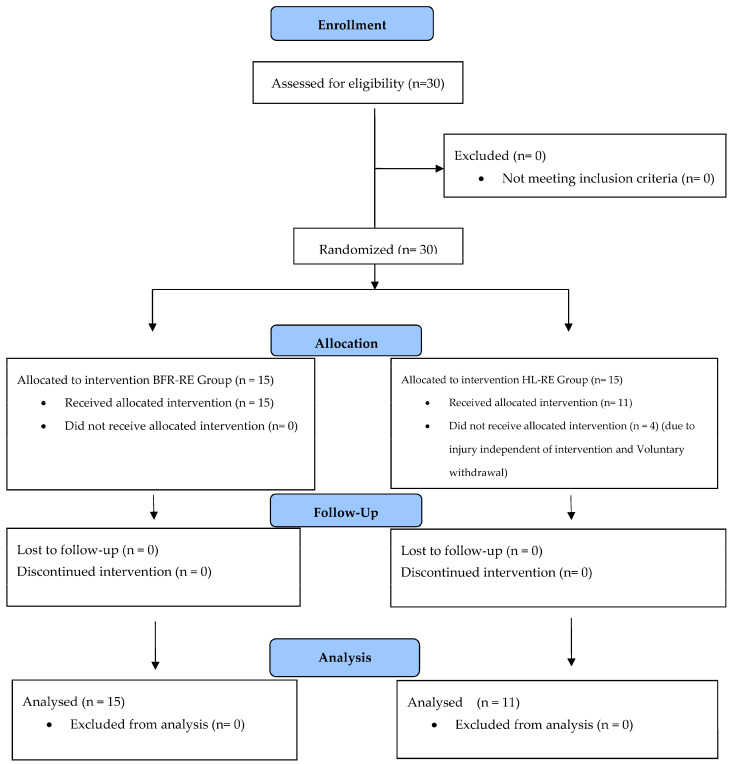
Consort flow diagram of this study. Legend. BFR-RE—blood flow restriction with resistance exercise; HL-RE—resistance exercise with high load.

The results of a previous study were used to determine the sample size [17]. G*Power software (version 3.1, University of Düsseldorf, Düsseldorf, Germany) was used to calculate the required sample size. Additionally, a priori power analysis was conducted using the following threshold values: repeated measures ANOVA, between factors, α = 0.05, β = 0.80, Cohen’s d = 0.50, number of groups = 2, number of measurements = 2, and r-value among repeated measures = 0.5. Although the minimum required sample size was 26, 30 participants were included in this study. Before enrollment, all participants received a comprehensive explanation of this study’s purpose, procedures, and potential risks. However, participants were unaware of the specific study groups to eliminate selection bias. The research protocol was approved by the Ordu University Non-Interventional Sports Sciences Research Ethics Committee (Code: 2024-05). This study adhered to the ethical guidelines outlined in the Declaration of Helsinki, and all participants provided informed consent before participation. Details regarding the participants’ characteristics and this study’s CONSORT flow diagram are presented in Table 1 and Figure 1.

#### 2.1.3. Procedures

**Figure 2:**
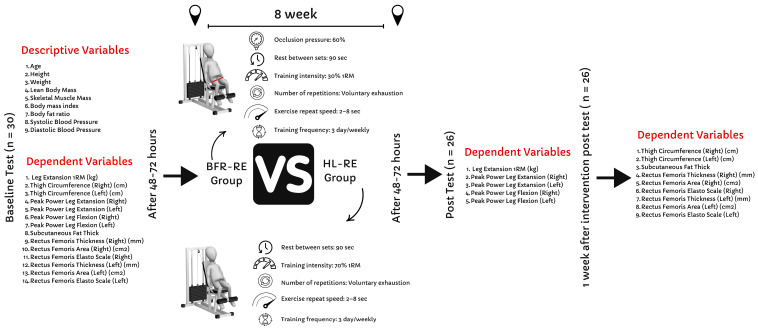
Experimental procedures of this study.

Random assignment was used to allocate participants to one of two intervention groups: (a) BFR-RE (*n* = 15) and (b) HL-RE (*n* = 15). Group allocation was performed with 1:1 allocation using an online randomization tool (Urbaniak & Plous, S. Research Randomizer Version 4.0; www.randomizer.org, accessed on 15 March 2025). The BFR-RE group performed bilateral leg extension exercises to volitional exhaustion, with a 90 s rest between sets at 30% of their one-repetition maximum (1RM) while wearing BFR cuffs set to 60% of limb occlusion pressure. The HL-RE group executed the same exercise to volitional exhaustion, with a 90 s rest between sets, but at 70% of their 1RM and without BFR cuffs. Participants were allowed 30 to 90 s of rest between sets, following the National Strength and Conditioning Association (NSCA) recommendations for hypertrophic adaptation [24]. The tempo for each repetition was established at 2–8 s for both the eccentric and concentric phases, as previous research has indicated that varying repetition tempos within this range can elicit similar hypertrophic responses [25]. A mobile application (Pro Metronome, Xanin Technology, Berlin, Germany) was used to monitor repetition tempos. Muscle strength and leg extension 1RM were assessed 48–72 h before and after the intervention. Muscle thickness, stiffness, subcutaneous fat thickness, and CSA were evaluated 48–72 h before and one-week post-intervention. The present study deemed a 7-day recovery period as appropriate, following the current evidence [21]. A standardized 10 min warm-up preceded each training session. Both groups performed bilateral leg extension exercises three times a week (on non-consecutive days) for eight weeks. The training load was adjusted weekly to ensure adherence to the progressive overload. Each participant’s 1RM was determined at the beginning of each week, and training sessions commenced 24 h later. Participants were instructed to refrain from strenuous physical exercise 48 h prior to the testing sessions. However, no specific nutritional recommendations were provided. The details of the study procedures and flow diagrams are presented in Figure 2.

### 2.2. Measurements

#### 2.2.1. Height, Weight, and Body Mass Index Measurements

Height, weight, and body mass index (BMI) were measured using a stadiometer (InBody 270, Biospace Inc., Cerritos, CA, USA) while subjects wore light clothing and no shoes. Measurements were taken before the exercise program.

#### 2.2.2. Blood Pressure and Heart Rate Measurements

After a 5 min supine rest, participants’ left brachial blood pressure (BP) was measured twice at 1 min intervals. The average of the two closest readings (within five mm Hg) was recorded in mm Hg [21]. Blood pressure and heart rate (HR) were assessed using a mercury sphygmomanometer (Comfort Plus DM-102 Perfect Aneroid, HONSUN Group, Danyang, Jiangsu, China).

#### 2.2.3. Determination of Limb Occlusion Pressure

Following a 10 min supine rest period, bilateral limb occlusion pressure (LOP) was assessed for both the right and left legs using a 13.5 cm pneumatic tourniquet (Reiser, Berlin, Germany). LOP was determined by progressively increasing the cuff pressure until the pulsation of the posterior tibial artery was no longer palpable. The point at which pulse oximetry (Choicemed MD300C12, ChoiceMMed, Seoul, Republic of Korea) on the hallux failed to detect oxygen saturation was recorded, along with the pulse rate, as indicative of 100% arterial occlusion [26]. After establishing the LOP, subjects prepared for the leg extension exercise by connecting the hose from the cuffs. The cuffs remained inflated throughout the exercise set and deflated immediately after completion [17].

#### 2.2.4. Determination of One-Repetition Maximum Strength

The 1RM leg extension test was conducted according to the criteria established by the researchers [27]. Participants were instructed to exert maximum effort throughout the test. A 5-to-10 min warm-up, which included dynamic stretching and running, was performed before the 1RM assessment. Participants utilized their full knee range of motion for each repetition. Following the warm-up, participants completed a preliminary set of 5 repetitions at 50% of their estimated 1RM. Subsequently, participants’ 1RM was determined over a maximum of five sets, with a minimum increase of 10% applied to the weight lifted in each subsequent set. Participants were allowed a rest period of 3 to 5 min between sets. A single session was utilized for each participant to ascertain their 1RM. The 1RM for all participants was established within the same week.

#### 2.2.5. Leg Extension and Flexion Peak Power Test

Before the peak power assessment, a familiarization period was conducted for participants using a Lafayette Manual Muscle Testing System (Lafayette Instrument Company, Lafayette, IN, USA). Subsequently, a professional strength and conditioning coach guided each participant through practice repetitions of leg extension and leg flexion movements until correct execution was confirmed. The order of the movements was randomized. Participants were then verbally instructed to perform three maximal contractions for each movement, with a 3 s hold for each contraction. A 1 min rest interval was incorporated between successive contractions, and a 3 min rest interval was implemented between different movement sets [28]. Leg extension was measured with participants in a seated position, while leg flexion measurements were taken with participants in a prone position. Each test was administered twice, and the participants’ highest scores were used for statistical analysis. Good reliability was observed between trials, and the details of the reliability analysis are presented in Table 2.

#### 2.2.6. Assessment of Thigh Circumference

Thigh circumference measurement was performed at 33% distal to the inguinal crease, precisely matching the intended cuff application site as verified by a trained investigator. The inguinal crease-to-superior patellar pole distance was recorded using a standard anthropometric tape measure [20]. Details regarding thigh circumference measurement are presented in Figure 2.

#### 2.2.7. Assessment of Muscle Thickness, Subcutaneous Fat Thickness, and Cross-Sectional Area

Sonographic measurements were conducted on the subjects after 48 to 72 h of rest without physical activity at baseline and one-week post-intervention. All measurements were performed using the Aplio 500 ultrasound device (Toshiba, Tokyo, Japan). Rectus femoris muscle thickness was assessed with a linear probe operating at a frequency of 12 MHz. For measuring the rectus femoris muscle CSA, a 3.5 MHz convex probe was utilized, as the muscle could not be fully captured within the field of view of the linear probe in a single cross-section. Measurements were taken in the axial plane at a 90-degree angle, ensuring the probe was in contact with the skin without applying pressure. Two radiologists with 14 and 7 years of experience performed all measurements concurrently, ensuring inter-rater reliability through consensus agreement. The greater trochanter and lateral femoral condyle were used as anatomical landmarks to standardize the measurement location. Muscle thickness and CSA of the rectus femoris were measured at the midpoint between these landmarks. Additionally, subcutaneous fat thickness at this precise location was also recorded. All measurements for these parameters were performed three times, and the mean values were reported. Details regarding thigh circumference measurement are presented in Figure 2.

#### 2.2.8. Assessments of Muscle Stiffness

Following the muscle thickness measurements and CSA measurements, the elasticity of the rectus femoris muscle was evaluated using the musculoskeletal strain elastography mode of the same ultrasound system and its linear probe. A five-point color scale was utilized, with the predominant color within the muscle determined by consensus between two radiologists and interpreted according to the scale. Red indicated high elasticity (soft tissue), while blue indicated low elasticity (stiff tissue); a score of 1 represented the highest elasticity, and a score of 5 represented the lowest [29,30]. Elastography measurements were obtained only once due to the inconsistent reproducibility of the measurement technique (e.g., varying compression forces, ultrasound settings). Elasto scores were determined by consensus between two radiologists. Elastography assessments were conducted by the same observers using the same technique 48 to 72 h before the commencement of the exercise program and one-week after.

#### 2.2.9. Statistical Analyses

The dataset for this study comprised 9 descriptive variables and 14 dependent variables (see Figure 1). A total of 5.1% of the data was missing from the descriptive variables due to participants, while 8.1% was missing from the dependent variables. Excluding participants with missing data could introduce selection bias and compromise randomization [31]. Therefore, intent-to-treat (ITT) analysis was employed in this study. To assess whether the missing data were randomly distributed across the groups, a missing completely at random (MCAR) test was conducted [32]. The results indicated that the data were randomly distributed among the groups (Chi-square = 4.55 to 31.93, df = 3 to 22, *p* = 0.07 to 0.20). Consequently, the missing data were imputed using the hot deck imputation method [33]. The usability of hot deck imputation on small samples was increased by the ranking according to related predictors method. Analyses were performed using frequentist and Bayesian statistical methods, and results were reported with descriptive statistics (mean ± standard deviation).

The normality of the distribution was assessed using skewness and kurtosis values for frequentist statistics. The reliability of muscle strength tests was calculated using the intraclass correlation coefficient (ICC), interpreted according to the following benchmarks: poor (>0.50), moderate (0.50 to 0.75), good (0.75 to 0.90), and excellent (0.90 to 1) [34]. A two (group) × two (time) repeated measures ANOVA was employed to analyze both main and interaction effects. Paired *t*-tests (for within-group comparisons) and independent *t*-tests (for between-group comparisons) were conducted, with Bonferroni correction applied for post hoc pairwise comparison analyses. Effect sizes were calculated using Cohen’s d and omega squared (ω^2^). Cohen’s d was interpreted according to the following reference intervals [35]: trivial (<0.2), small (0.20–0.59), medium (0.60–1.19), large (1.20–1.99), and very large (>2). Similarly, omega squared was interpreted based on reference intervals of 0.01 (small effect), 0.06 (moderate effect), and 0.14 (large effect) [36]. In all analyses for frequentist statistics, the significance level was set at α = 0.05.

While comparative hypothesis tests are performed with the arithmetic mean, standard deviation, and sample size of the groups, hypothesis tests can be affected by small sample sizes and sample numbers. However, Bayesian statistics can offer more flexible and adaptable analyses in complex models and provide the opportunity to comment directly on the probability distributions of the parameters [37]. Therefore, Bayesian statistics were employed in this study’s analyses as an alternative to frequentist statistics. A Bayesian 2 (group) × 2 (time) repeated measures ANOVA was conducted to evaluate both main and interaction effects. Bayesian paired sample and independent sample *t*-tests were utilized for post hoc analyses. The results of the analyses were reported using the Bayes factor for hypothesis testing (BF10) and the Bayes factor for inclusion in model comparison (BFincl). The strength of evidence for hypothesis H1 was interpreted based on the following criteria [38]: extreme (BF10 and BFincl > 100), very strong (BF10 and BFincl = 30 to 100), strong (BF10 and BFincl = 10 to 30), moderate (BF10 and BFincl = 3 to 10), and anecdotal (BF10 and BFincl = 1 to 3). For hypothesis H0, the following criteria were considered for assessing the strength of evidence [38]: extreme (BF10 and BFincl < 0.001), very strong (BF10 and BFincl = 0.001 to 0.03), strong (BF10 and BFincl = 0.03 to 0.01), moderate (BF10 and BFincl = 0.01 to 0.3), and anecdotal (BF10 and BFincl = 0.3 to 1). Statistical analyses were performed using JASP (version 0.19.1.0, Amsterdam, The Netherlands) and R software (version 4.1.0, R Core Team, Vienna, Australia). The {naniar}, {mvnmle}, {VIM}, and {ggplot2} packages were used for ITT analysis. The R code lines are presented through OSF (https://osf.io/mw2hn/).

## 3. Results

### 3.1. Results of Baseline Tests, and Adherence to Intervention

**Figure 3:**
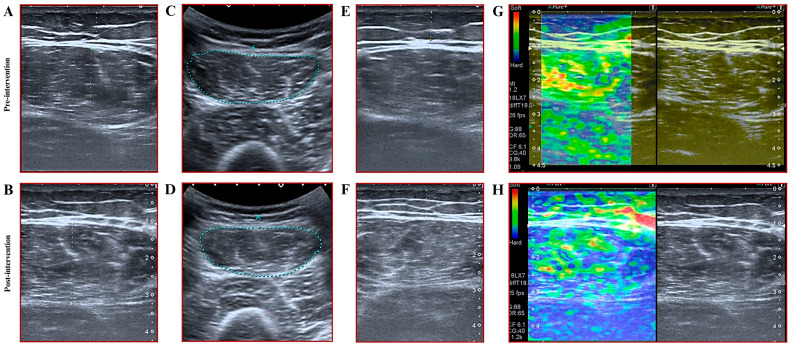
Muscle Thickness, muscle stiffness, subcutaneous fat tissue, and cross-sectional area assessments. Legend. (**A**): rectus femoris muscle pre-test thickness; (**B**): rectus femoris muscle post-test thickness; (**C**): rectus femoris muscle pre-test area; (**D**): rectus femoris muscle post-test area; (**E**): subcutaneous fat tissue pre-test; (**F**): subcutaneous fat tissue post-test; (**G**): rectus femoris elasto scale pre-test; (**H**): rectus femoris elasto scale post-test. (**A**,**B**): the thickness of the rectus femoris muscle is indicated by dot-shaped white lines, and is measured in mm. (**C**,**D**): the rectus femoris muscle cross-sectional area is shown with dotted blue lines, and is measured in cm^2^. (**E**,**F**) The thickness of subcutaneous fat tissue is marked by dotted white lines, and is measured in cm. (**G**,**H**): elastography color scale of the rectus femoris muscle of participants before (**G**) and after (**H**) exercise.

Baseline tests revealed no statistically significant differences in anthropometric measurements between the groups (*p* = 0.15 to 0.69) (see Table 1). Similarly, no statistically significant differences were observed between the BFR-RE and HL-RE groups in baseline tests for 14 dependent variables (*p* = 0.06 to 0.90) (see Figure 3A). Two participants from the HL-RE group withdrew from this study due to injuries unrelated to the intervention, and two participants voluntarily withdrew. Adherence to the intervention was 87.11% among all participants. Weekly training attendance schedules, reflecting participants’ adherence to the intervention, are presented via OSF (https://osf.io/mw2hn/).

### 3.2. Results of 1RM Muscle Strength and Muscle Power Tests

The main effect of time was statistically significant for peak power leg extension left (PPLEL), peak power leg extension right (PPLER), and peak power leg flexion right (PPLFR) (*p* = 0.01, ω^2^ = 0.148 to 0.343, BFincl = 68.00 to 4.968 × 10^+7^). Within-group comparisons revealed that an 8-week resistance exercise program significantly increased PPLEL, PPLER, and PPLFR performance in both groups (*p* = 0.01 to 0.04, d = 0.53 to 1.71, BF10 = 3.23 to 61,368.63). However, the effect of time on peak power leg flexion left (PPLFL) was not statistically significant (*p* = 0.15, ω^2^ = 0.011, BFincl = 0.99). The interaction between group and time was statistically insignificant for PPLER and PPLFR (*p* = 0.06 to 0.07, ω^2^ = 0.011 to 0.0114, BFincl = 1.75 to 2.00). Conversely, it was statistically significantly different for PPLEL and PPLFL (*p* = 0.01, ω^2^ = 0.020 to 0.050, BFincl = 3.05 to 7.65). Intergroup comparisons based on post-test results indicated a statistically significant difference in PPLFL performance in favor of the BFR-RE group (*p* = 0.02). However, there were no statistically significant differences between the groups for PPLEL, PPLER, and PPLFR performance (*p* = 0.54 to 0.96).

On the other hand, the results for 1RM performance indicated that the main effect of time was statistically significant (*p* = 0.01, ω^2^ = 0.52, BFincl = 7.886 × 10^+10^). Within-group comparisons revealed that both groups improved 1RM performance after 8 weeks of resistance exercise (*p* = 0.01, d = 1.97 to 2.21, BF10 = 1562.85 to 170,902.68). However, the interaction between group and time was not statistically significant (*p* = 0.47, ω^2^ = 0.000, BF10 = 0.57). For between-group comparisons based on the post-test, there was no statistically significant difference in 1RM performance between the BFR-RE group and the HR-LE group (*p* = 0.82).

Bayesian statistical results supported frequentist statistical results except for two analyses. Depending on whether the *p*-value was significant or not, hypotheses were supported by Bayesian evidence levels from anecdotal to extreme levels. Two analyses (PPLFR and PPLER) based on group × time interaction were not found significant in frequentist statistics. Bayesian statistics reported anecdotal evidence in favor of hypothesis H1. Analysis details for peak power and 1RM tests are presented in Table 3 and Figure 3B.

### 3.3. Results for Thigh Circumference and Subcutaneous Fat Thickness

For thigh circumference measurements (both left and right), the main effect of time was not statistically significant (*p* = 0.08 to 0.38, ω^2^ = 0.000 to 0.014, BFincl = 0.33 to 0.86). The group × time interaction did not have statistical significance (*p* = 0.06 to 0.85, ω^2^ = 0.000 to 0.010, BFincl = 0.27 to 0.59). Within-group comparisons also revealed statistically nonsignificant results (*p* = 0.43 to 1.00, d = 0.13 to 0.33, BF10 = 0.32 to 0.51), as did between-group comparisons based on post-test results (*p* = 0.62 to 0.70). Consequently, neither BFR-RE nor HL-RE training significantly improved thigh circumference over the eight weeks.

On the other hand, the results for subcutaneous fat thickness indicated that the main effect of time was statistically significant (*p* = 0.08, ω^2^ = 0.002, BFincl = 2.48). Within-group comparisons revealed a statistically significant difference in favor of BFR-RE (*p* = 0.01, d = 0.32, BF10 = 45.33). In contrast, no statistically significant difference was observed in the HL-RE group (*p* = 1.00, d = 0.09, BF10 = 0.43). Additionally, there was no statistically significant difference in subcutaneous fat thickness for the group × time interaction (*p* = 0.01, ω^2^ = 0.010, BFincl = 20.42) and between-group comparisons based on post-test results (*p* = 0.17).

Except for the analysis based on the group × time interaction of subcutaneous fat thickness, the results from Bayesian and frequentist statistics were consistent. The findings were supported by varying levels of evidence certainty, ranging from anecdotal to extreme, depending on the significance of the *p*-value. While the frequentist statistics did not indicate a significant difference in the group × time interaction of subcutaneous fat thickness, the Bayesian statistics suggested that there might be anecdotal evidence supporting the alternative hypothesis (H1). Detailed analyses of thigh circumference and subcutaneous fat thickness are presented in Table 3 and Figure 3B.

### 3.4. Results for Muscle Thickness, Cross-Sectional Area, and Muscle Stiffness

For rectus femoris thickness on the left (RFTL), rectus femoris thickness on the right (RFTR), rectus femoris cross-sectional area on the left (RFCSAL), rectus femoris cross-sectional area on the right (RFCSAR), rectus femoris stiffness on the left (RFSL), and rectus femoris stiffness on the right (RFSR), the main effect of time demonstrated statistically significant differences (*p* = 0.01, ω^2^ = 0.211 to 0.411, BFincl = 209.15 to 3.928 × 10^+9^). Within-group comparisons revealed statistically significant results favoring both the BFR-RE and HL-RE groups across five variables (RFTR, RFTL, RFCSAR, RFCSAL, RFSL) (*p* = 0.01, d = 0.82 to 1.70, BF10 = 14.96 to 8766.54). Conversely, within-group comparisons for RFSR indicated a significant difference favoring the BFR-RE group (*p* = 0.04, d = 1.07, BF10 = 5.37). In contrast, no significant difference was observed for the HL-RE group (*p* = 0.12, d = 1.06, BF10 = 2.11). For six variables (RFTR, RFTL, RFCSAR, RFCSAL, RFSL, RFSR), the group × time interaction was not statistically significant (*p* = 0.13 to 0.98, ω^2^ = 0.000 to 0.007, BFincl = 0.54 to 0.94). Between-group comparisons based on post-test results revealed statistically non-significant differences between the BFR-RE and HL-RE groups for the six variables (RFTR, RFTL, RFCSAR, RFCSAL, RFSL, RFSR) (*p* = 0.32 to 0.80).

**Figure 4:**
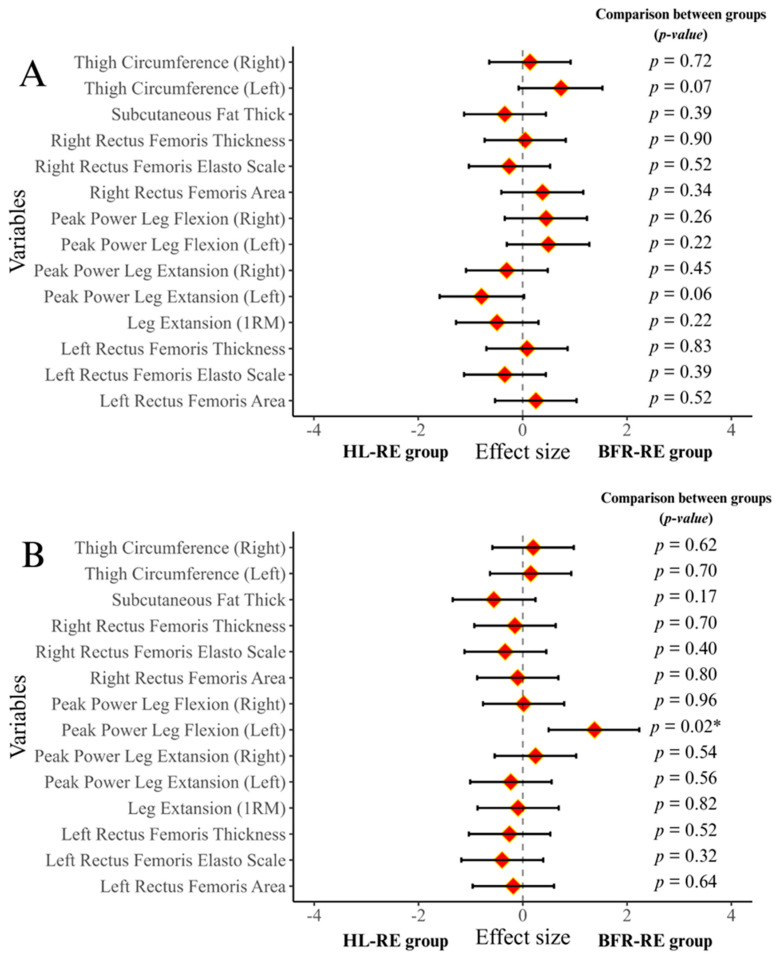
Forest plots based on pre- and post-tests of experimental groups. Legend. (**A**): pre-test; (**B**): post-test. BFR-RE—blood flow restricted training; HL-RE—high-load resistance exercise; *: *p* < 0.05. In this figure, *p*-values include the comparisons of the groups in the pre-test and post-test. While the comparison results are reported with *p*-values, the results with significant differences are indicated by *. The positions of the red diamonds in the figure indicate the effect size in favor of that group.

The results of the frequentist statistical analysis were corroborated by those of the Bayesian statistical analysis, except for the within-group comparison for HL-RE in the RFSR variable. The findings were supported by varying levels of certainty, ranging from anecdotal to extreme, depending on the significance of the *p*-value. While the frequentist analysis indicated a statistically insignificant difference in the within-group comparisons of HL-RE in the RFSR variable, the Bayesian analysis suggested the presence of anecdotal evidence in favor of the alternative hypothesis (H1). Detailed information regarding muscle thickness and CSA analyses is presented in Table 3 and Figure 3 and Figure 4.

## 4. Discussion

The effects of BFR-RE and HL-RE on quadriceps muscle adaptation were investigated in this study. After group allocation, four participants voluntarily withdrew from the HL-RE group before the interventions commenced. To maintain the integrity of the randomization process, the researchers considered the participants’ initial group assignments for evaluation [31]. According to the power analysis, since the minimum sample size required was 26 participants, the 4 withdrawals did not compromise the statistical power of this study.

The findings demonstrated comparable improvements in quadriceps muscle adaptations following BFR-RE and HL-RE interventions. Significant increases were observed in the 1RM, muscle strength, thickness, CSA, stiffness, and subcutaneous fat thickness, measured from baseline to follow-up in both groups. To our knowledge, this study is the first to compare the effects of BFR-RE and HL-RE on quadriceps adaptations one week post-intervention, utilizing a training protocol emphasizing voluntary exhaustion in untrained men. We hypothesized that there would be no statistically significant difference in hypertrophic responses between the BFR-RE and the HL-RE groups in all parameters or that significant results would favor BFR-RE. The results of this study supported this hypothesis, demonstrating comparable effectiveness between low-load BFR-RE and traditional HL-RE across outcome measures. Frequentist and Bayesian approaches largely supported each other, but some results were contradictory. This may be because frequentist statistics are affected by the number of samples. Researchers emphasized that Bayesian statistics provide more information than frequentist statistics and are more sensitive in studies with limited data [39].

Analysis revealed statistically significant improvements in 1RM following both BFR-RE and HL-RE interventions. Specifically, the magnitude of the increase in 1RM strength was 44.31% in the BFR-RE group and 36.91% in the HL-RE group. These observed improvements in 1RM are consistent with prior research demonstrating the effectiveness of both BFR-RE and HL-RE training modalities. However, unlike our present study where sets were performed to voluntary exhaustion, the prior investigations often employed a 30-15-15-15 repetition scheme. These studies reported 1RM increases of 17.88% with BFR-RE and 20.63% with HL-RE [17]. This finding suggests that utilizing BFR with sets taken to voluntary exhaustion may elicit more pronounced improvements in 1RM strength. Our findings align with previous research [17,40], indicating that BFR-RE can elicit 1RM gains comparable to HL-RE despite utilizing lower training loads. Consequently, both BFR-RE and HL-RE may serve as effective interventions for enhancing 1RM in untrained individuals.

No statistically significant main effect of time, group × time interaction, within-group changes, or between-group differences were observed for bilateral thigh circumference. Neither BFR-RE nor HL-RE training significantly improved bilateral thigh circumference after the eight-week intervention. Muscle growth following RE can result from the accumulation of contractile proteins and ECF associated with muscle damage [23]. Contrary to previous findings [17,41], the lack of change in thigh circumference may be attributed to the timing of post-intervention measurements in the present study. Unlike prior research, thigh circumference was assessed one week post-intervention, allowing sufficient time for edema resolution, which may account for the decrease observed in the left thigh. The influence of performing thigh circumference assessments 4–5 days after the final training session, as recommended in the literature [21], on the study outcomes should be considered.

Regarding PPLE, both groups demonstrated significant improvements, supported by a notable main effect of time. The percent change in PPLER was 25.95% in the BFR-RE group and 16.79% in the HL-RE group. PPLEL increased by 19.42% in the BFR-RE group and 8.30% in the HL-RE group. The results suggest that the leg extension training program led to enhancements specific to the targeted muscle group without resulting in concomitant gains in PPLF. This underscores the importance of designing targeted training programs to achieve specific outcomes [17].

A noteworthy finding of this study was that between-group comparisons at the post-intervention assessment revealed a statistically significant difference in PPLF performance, favoring the BFR-RE group. The significant improvements in PPLF observed in the BFR-RE group may be attributable to the specific training protocol employed in this study or to the relatively short duration of the intervention [17]. These findings corroborate previous research demonstrating the efficacy of BFR-RE and HL-RE in enhancing muscle strength across diverse populations [16,42]. No significant difference in leg extension strength was observed between the BFR-RE and HL-RE groups. This suggests that both training protocols were equally effective in enhancing leg extension strength when performed to volitional failure. These findings are consistent with previous research demonstrating comparable improvements in muscle strength following both BFR-RE and HL-RE interventions [16,43].

A statistically significant main effect of time was observed for subcutaneous fat thickness. Within-group comparisons revealed a statistically significant reduction in subcutaneous fat thickness only in the BFR-RE group. No statistically significant group × time interaction effect or between-group differences in subcutaneous fat thickness at the post-intervention assessment were observed. The observed reduction in subcutaneous fat thickness suggests a potential advantage of the BFR-RE intervention compared with HL-RE. This reduction in subcutaneous fat may yield notable improvements in athletic performance, body composition, metabolic health, and cardiovascular health.

Potential mechanisms warranting investigation include hormonal alterations, increased energy expenditure, and other contributing factors. While no prior research has investigated the effects of BFR-RE on subcutaneous fat thickness, the extant literature includes studies employing analogous mechanisms that inform our understanding of potential effects. Recent research indicates that resistance training and hypoxia may represent a beneficial strategy for reducing subcutaneous adipose tissue and improving body composition via modulation of metabolic cytokine levels [44]. BFR indirectly influences cellular metabolism by inducing metabolic byproduct accumulation while concurrently restricting blood flow in the proximal limb segment, thereby simulating a localized hypoxic environment [45]. Combined resistance training and hypoxia may promote greater fat loss than either intervention alone. Hypoxic conditions may shift the body’s metabolic preference towards lipid oxidation for energy, thereby increasing fat mobilization from adipose tissue. This metabolic adaptation is further supported by enhanced mitochondrial biogenesis and improved capillary density, leading to greater fat utilization during resistance exercise [46].

Regarding hypertrophy outcomes, our findings demonstrate that both BFR-RE and HL-RE significantly increased rectus femoris muscle thickness in both the left and right legs. The notable increases in rectus femoris muscle thickness observed in both groups (BFR-RE: RFTR 16.16%, RFTL 15.08%; HL-RE: RFTR 19.03%, RFTL 19.20%) indicate that both training modalities effectively induced muscle hypertrophy during the intervention. These findings align with previous research [16,40,41,47], showing that BFR-RE and HL-RE promote muscle growth across various populations and muscle groups. However, in contrast to the present study, the sets were not performed to volitional failure in any of these prior investigations.

A recent study, employing a six-week training protocol of bilaterally lying leg curl exercises (performed two days per week using a 30-15-15-15 repetition scheme), reported a 10.86% increase in semimembranosus muscle thickness in the right leg for the BFR-RE group, compared with 11% in the HL-RE group [17]. When contrasted with these results, the findings of our study suggest that performing both BFR-RE and HL-RE to volitional failure may be a more effective approach for inducing muscle hypertrophy. The non-significant main effect of the group and the absence of a statistically significant group × time interaction suggest that, despite substantial improvements in muscle thickness, no significant difference in muscle hypertrophy existed between BFR-RE and HL-RE. The results demonstrate the equivalent efficacy of both training methods in stimulating quadriceps hypertrophy when performed to voluntary exhaustion.

The observation of similar hypertrophic gains with low-load BFR-RE and HL-RE may be attributed to several factors, one of which is the primary mechanism by which low-load BFR training promotes muscle hypertrophy, i.e., increased metabolic stress. Studies have shown that low-load resistance exercises combined with BFR lead to significant metabolic accumulation, a key stimulus for muscle growth. For instance, Suga et al. demonstrated that metabolic stress during low-intensity resistance exercise with BFR was markedly elevated compared with high-load training, suggesting that metabolic byproducts play a crucial role in hypertrophic signaling pathways [48]. Additionally, Bielitzki found that low-load BFR training increased deoxyhemoglobin concentration, a marker of metabolic stress, compared with high-load training [49]. This accumulation of metabolites, such as lactate, activates anabolic signaling pathways, including the mechanistic target of the rapamycin (mTOR) pathway, which is essential for muscle protein synthesis [50]. These findings underscore the importance of BFR as a legitimate training modality for muscle hypertrophy. For example, post-exercise increases in protein synthesis of 40–50% have been observed with BFR protocols, potentially due to mTOR pathway activation [51]. This heightened response can be advantageous for individuals unable to tolerate high-intensity training, including older adults and those in rehabilitation.

While this study demonstrates that low-load BFR training can produce hypertrophic effects comparable to high-load resistance training, potentially through mechanisms such as increased metabolic stress and hypoxic adaptations, the literature includes studies questioning the direct role of metabolic stress in driving hypertrophy. While the present findings suggest that BFR training may support hypertrophic adaptations at lower loads, uncertainty remains regarding metabolic stress’s independent direct effect on hypertrophy. Therefore, further research is needed to elucidate the independent contribution of metabolic stress to hypertrophic gains.

CSA is a critical determinant of muscle mass, and its modification directly influences muscle function [52,53] while exhibiting high predictive validity for muscle force production capacity [54]. The present study revealed a statistically significant main effect of time for both the RFCSAR and RFCSAL variables (respectively, BFR-RE: RFSCAR, 17.68%, RFCSAL, 29.04%, HL-RE: RFCSAR, 15.81%, RFCSAL, 25.17%). Within-group analyses demonstrated statistically significant improvements in both variables for the BFR-RE and HL-RE groups. However, no statistically significant group × time interaction effect was observed for RFCSAR or RFCSAL. These findings indicate that BFR-RE is equally effective as HL-RE in promoting increases in CSA among untrained males.

Our results are consistent with a recent study that reported comparable hypertrophic responses in well-trained individuals following eight weeks of training to voluntary exhaustion, regardless of the repetition range (25–35 repetitions vs. 8–12 repetitions) [55]. Furthermore, our findings support the established understanding that blood flow restriction applied during low-load resistance training effectively increases muscle CSA [56]. Despite the absence of a 7-day post-training recovery period in the reviewed studies, remarkably similar results were observed, in contrast to the current study.

While ultrasonography has been widely used for the safe and practical assessment of muscle stiffness [57], no studies have investigated quadriceps muscle stiffness one week following BFR-RE intervention. Assessment of quadriceps muscle stiffness following injury may prove valuable in guiding rehabilitation strategies for athletes and sedentary individuals, providing insight into injury risk associated with elevated stiffness and reductions in stiffness following injury prevention programs. In this context, the present study employed ultrasonography to assess quadriceps muscle stiffness following BFR-RE and HL-RE interventions.

Our analysis revealed a statistically significant main effect of time for both RFSL and RFSR. Within-group comparisons showed statistically significant improvements in RFSL for both the BFR-RE and HL-RE groups. In contrast, within-group comparisons for RFSR revealed a significant improvement only in the BFR-RE group, with no significant change observed in the HL-RE group. The group × time interaction effect was not statistically significant for either RFSL or RFSR. Finally, between-group comparisons at the post-intervention assessment revealed no statistically significant differences in RFSL or RFSR between the BFR-RE and HL-RE groups.

The observation that only the BFR-RE group exhibited a significant improvement in RFSR while the HL-RE group showed no change warrants further investigation. Potential contributing factors include the unique physiological mechanisms of BFR-RE, such as metabolic stress [58] and altered blood flow dynamics, which may differentially impact muscle stiffness. HL-RE, lacking this ischemic component, might produce strength gains without the same influence on tissue stiffness. This could be further explored by including the relevant literature on the effects of ischemia on connective tissue properties. Finally, limitations of the study design, namely the reliance on self-reported exertion levels, may have attenuated the observed changes in stiffness.

Also, the between-group comparisons conducted at the post-intervention assessment revealed no statistically significant differences in RFSL or RFSR between the BFR-RE and HL-RE groups. The finding that only the BFR-RE group demonstrated a significant improvement in RFSR, while the HL-RE group exhibited no change, warrants further investigation. Potential contributing factors include the unique physiological mechanisms associated with BFR-RE, such as metabolic stress and altered blood flow dynamics, which may differentially affect muscle stiffness. In contrast, HL-RE, which lacks this ischemic component, might yield strength gains without exerting the same influence on tissue stiffness. This aspect could be further explored by incorporating relevant literature on the effects of ischemia on connective tissue properties.

Although both groups experienced training until voluntary exhaustion, there may have been subtle differences in overall repetition that influenced stiffness adaptations. This could be further investigated by comparing the total number of repetitions performed by each group and examining the relationship between total number of repetitions and changes in muscle stiffness within the existing literature. Lastly, limitations of the study design, particularly the reliance on self-reported exertion levels, may have attenuated the observed changes in stiffness.

As observed in our study and reported in the literature, aligning with our findings, BFR-RE with low-load strength training can offer several distinct advantages (hypertrophy, strength, etc.) over traditional resistance training, particularly for individuals who may not tolerate high loads, such as athletes in rehabilitation, older adults, post-surgical patients, or those with specific medical conditions [43]. However, it is worth noting that, in the traditional model, individuals can train independently, whereas this method may necessitate the assistance of a professional, at least for a certain period, in addition to the discomfort associated with the blood flow restriction technique. Nevertheless, despite these potential drawbacks, this methodology is considered to be a highly valuable training tool for coaches, strength and conditioning specialists, and clinicians.

Several potential limitations of this study, beyond those already addressed, should be acknowledged. The BFR technique employed, while potentially effective, may have presented discomfort to participants, including the risk of pain and potential fear of vascular complications, which might have influenced their performance and/or willingness to cooperate. Also, the small sample size, potential placebo effects, and the absence of biochemical/hormonal measurements should be considered. These concerns need consideration in interpreting the results.

## 5. Conclusions

This study investigated the effectiveness of BFR-RE versus HL-RE on hypertrophic adaptations in untrained young males. The results demonstrated comparable increases in 1RM strength and muscle thickness, CSA, and stiffness following both protocols. While BFR-RE showed a slightly greater effect on some variables (e.g., rectus femoris stiffness and subcutaneous fat thickness), the overall findings suggest that BFR-RE may be a viable alternative to HL-RE for promoting muscle hypertrophy in untrained individuals, particularly in situations where high-load resistance training is not feasible or desirable. Further research is warranted to explore the optimal parameters of BFR-RE protocols and the underlying mechanisms of these effects.

## Figures and Tables

**Table 1:** Descriptive characteristics of the participants.

Variables/Groups	BFR-RE Group (*n* = 15)	HL-RE Group (*n* = 11)	*p*-Value
	(Mean ± SD)	(Mean ± SD)	
Age (years)	22.13 ± 3.29	20.72 ± 1.73	0.21
Height (cm)	180.93 ± 7.88	177.54 ± 7367	0.27
Weight (kg)	75.50 ± 12.39	71.74 ± 8.48	0.39
Lean Body Mass (kg)	63.16 ± 8.83	59.70 ± 6.10	0.27
Skeletal Muscle Mass (kg)	35.84 ± 5.33	33.87 ± 3.54	0.29
Body Mass Index (%)	23.04 ± 3.35	22.57 ± 2.48	0.69
Body Fat Ratio (%)	15.92 ± 5.49	16.50 ± 5.46	0.79
SBP (mm Hg)	115.33 ± 7.43	120.00 ± 8.94	0.15
DBP (mm Hg)	76.00 ± 7.36	78.18 ± 6.03	0.43
Right LOP (mm Hg)	157.86 ± 19.97	Not applied	N/A
Left LOP (mm Hg)	160.36 ± 20.39	Not applied	N/A

Legend. BFR-RE—resistance training with blood flow restriction; HL-RE—high-load resistance training; SBP—systolic blood pressure; DBP—diastolic blood pressure; LOP—limb occlusion pressure.

**Table 2:** Interclass correlation coefficient results regarding maximal repeat strength tests.

Variables	ICC	95%CI Lower	95%CI Upper	Reliability Interpretation
Peak Power Leg Extansion Pre-Test (Right)	0.75	0.44	0.89	Good
Peak Power Leg Extansion Post-Test (Right)	0.82	0.60	0.92	Good
Peak Power Leg Extansion Pre-Test (Left)	0.83	0.64	0.92	Good
Peak Power Leg Extansion Post-Test (Left)	0.87	0.70	0.95	Good
Peak Power Leg Flexion Pre-Test (Right)	0.83	0.62	0.93	Good
Peak Power Leg Flexion Post-Test (Right)	0.87	0.70	0.95	Good
Peak Power Leg Flexion Pre-Test (Left)	0.83	0.61	0.93	Good
Peak Power Leg Flexion Post-Test (Left)	0.84	0.65	0.93	Good

Legend. ICC—interclass correlation coefficient; CI—confidence interval.

**Table 3:** Results on the effects of blood flow restriction versus traditional strength training on muscle hypertrophy.

Variables	Groups	Pre-Test	Post-Test		Frequentist RM-ANOVA						Bayesian RM-ANOVA							
					Intra-Group Comparisons		Time (Main Effect)		Group × Time (İnteraction)		Intra-Group Comparisons			Time (Main Effect)			Group × Time (İnteraction)	
		Mean ± SD	Mean ± SD		*p*-Value (Cohen’d)		*p*-Value (Omesquare)		*p*-Value (Omegasquare)		H	BF_10_ (Level)		H	BF_incl_ (Level)		H	BF_incl_ (Level)
Leg Extansion 1RM (kg)	BFR-RE	111.58 ± 16.42	161.03 ± 31.92		0.01 * (2.21)		0.01 * (0.52)		0.479 (0.000)		H1	170902.68 (extreme)		H1	7.886 × 10^+10^ (extreme)		H0	0.57 (anecdotal)
	HL-RE	119.42 ± 15.33	163.50 ± 18.58		0.01 * (1.97)						H1	1562.85 (extreme)						
Thigh Circumference (Right) (cm)	BFR-RE	54.60 ± 4.77	55.93 ± 4.87		0.81 (0.31)		0.08 (0.014)		0.857 (0.000)		H0	0.51 (anecdotal)		H0	0.86 (anecdotal)		H0	0.27 (anecdotal)
	HL-RE	54.00 ± 3.37	55.09 ± 3.08		1.00 (0.25)						H0	0.35 (anecdotal)						
Thigh Circumference (Left) (cm)	BFR-RE	56.20 ± 4.61	55.60 ± 6.23		1.00 (0.13)		0.38 (0.000)		0.06 (0.010)		H0	0.32 (anecdotal)		H0	0.33 (anecdotal)		H0	0.59 (anecdotal)
	HL-RE	53.27 ± 2.86	54.81 ± 3.02		0.43 (0.33)						H0	0.37 (anecdotal)						
Peak Power Leg Extansion (Right)	BFR-RE	28.55 ± 4.61	35.96 ± 3.69		0.01 * (1.71)		0.01 * (0.343)		0.06 (0.014)		H1	61368.63 (extreme)		H1	4.968 × 10^+7^ (extreme)		H1	1.75 (anecdotal)
	HL-RE	29.95 ± 4.56	34.98 ± 4.43		0.01 * (1.16)						H1	132.59 (extreme)						
Peak Power Leg Extansion (Left)	BFR-RE	24.86 ± 3.41	29.96 ± 3.52		0.01 * (1.15)		0.01 * (0.151)		0.01 * (0.020)		H1	8359.30 (extreme)		H1	68.00 (extreme)		H1	7.65 (moderate)
	HL-RE	28.54 ± 6.01	30.91 ± 4.93		0.03 * (0.53)						H1	5.34 (moderate)						
Peak Power Leg Flexion (Right)	BFR-RE	23.84 ± 2.71	21.28 ± 2.04		0.01 * (1.08)		0.01 * (0.148)		0.07 (0.011)		H1	2317.33 (extreme)		H1	99.00 (extreme)		H1	2.00 (anecdotal)
	HL-RE	22.63 ± 2.65	21.25 ± 1.83		0.04 * (0.58)						H1	3.23 (moderate)						
Peak Power Leg Flexion (Left)	BFR-RE	20.91 ± 1.26	21.21 ± 1.74		1.00 (0.21)		0.15 (0.011)		0.01 * (0.050)		H0	0.33 (anecdotal)		H0	0.99 (anecdotal)		H1	3.05 (moderate)
	HL-RE	20.25 ± 1.42	19.14 ± 1.07		0.08 (0.78)						H1	5.82 (moderate)						
Subcutaneous Fat Thick (cm)	BFR-RE	5.65 ± 1.82	5.01 ± 1.87		0.01 * (0.32)		0.01 * (0.010)		0.08 (0.002)		H1	45.33 (extreme)		H1	20.42 (strong)		H1	2.48 (anecdotal)
	HL-RE	6.32 ± 2.15	6.13 ± 2.20		1.00 (0.09)						H0	0.43 (anecdotal)						
Rectus Femoris Thickness (Right) (mm)	BFR-RE	20.04 ± 3.16	23.28 ± 3.41		0.01 * (1.19)		0.01 * (0.298)		0.44 (0.000)		H1	2330.98 (extreme)		H1	1.588 × 10^+7^ (extreme)		H0	0.63 (anecdotal)
	HL-RE	19.91 ± 1.52	23.70 ± 1.56		0.01 * (1.40)						H1	990.22 (extreme)						
Rectus Femoris CSA (Right) (cm^2^)	BFR-RE	8.20 ± 1.86	9.65 ± 2.16		0.01 * (0.85)		0.01 * (0.225)		0.15 (0.007)		H1	159.74 (extreme)		H1	222.00 (extreme)		H0	0.94 (anecdotal)
	HL-RE	7.61 ± 1.02	9.82 ± 1.11		0.01 * (1.30)						H1	50.54 (extreme)						
Rectus Femoris Stiffness (Right)	BFR-RE	2.80 ± 0.86	3.53 ± 0.64		0.04 * (1.07)		0.01 * (0.213)		0.98 (0.000)		H1	5.37 (moderate)		H1	209.15 (extreme)		H0	0.46 (anecdotal)
	HL-RE	3.00 ± 0.63	3.72 ± 0.46		0.12 (1.06)						H1	2.11 (anecdotal)						
Rectus Femoris Thickness (Left) (mm)	BFR-RE	19.03 ± 2.61	21.90 ± 2.63		0.01 * (1.32)		0.01 * (0.360)		0.13 (0.004)		H1	60172.78 (extreme)		H1	3.928 × 10^+9^ (extreme)		H0	0.97 (anecdotal)
	HL-RE	18.85 ± 1.04	22.47 ± 1.49		0.01 * (1.66)						H1	8766.54 (extreme)						
Rectus Femoris CSA (Left) (cm^2^)	BFR-RE	7.65 ± 1.63	8.86 ± 1.80		0.01 * (0.82)		0.01 * (0.211)		0.18 (0.005)		H1	230.87 (extreme)		H1	8849.34 (extreme)		H0	0.79 (anecdotal)
	HL-RE	7.31 ± 0.79	9.15 ± 1.21		0.01 * (1.25)						H1	22.41 (strong)						
Rectus Femoris Stiffness (Left)	BFR-RE	2.46 ± 0.74	3.60 ± 0.63		0.01 * (1.70)		0.01 * (0.411)		0.89 (0.000)		H1	668.61 (extreme)		H1	1.232 × 10^+6^ (extreme)		H0	0.54 (anecdotal)
	HL-RE	2.72 ± 0.78	3.81 ± 0.40		0.01 * (1.64)						H1	14.96 (strong)						

Legend. RM-ANOVA: Repested measures ANOVA; SD: Standard deviation; BFR-RE: Resistance exercise with blood flow restriction; HL-RE: Resistance exercises with high load; H: Supported hypothesis. Numbers too large to be reported in the table are multiplied by 10 and reported by multiples of 10. For example, 7.886 × 10^+10^ represents 78,860,000,000 (i.e., 78.86 billion); *: Bold and asterisked numbers indicate statistically significant results.

## Data Availability

Open access to the data is presented available through OSF (https://osf.io/mw2hn/).

## Supplementary Materials

The following supporting information can be downloaded at: https://www.mdpi.com/article/10.3390/medicina61050804/s1.

## Abbreviations

BFR-RE	Resistance exercise with blood flow restriction
HL-RE	High-load resistance exercise
EMG	Electromyographic
CSA	Cross-sectional area
1RM	One-repetition maximum
NSCA	National Strength and Conditioning Association
ECF	Extracellular fluid
MRI	Magnetic resonance imaging
ITT	Intent-to-treat
MCAR	Missing completely at random
ICC	Intraclass correlation coefficient
PPLEL	Peak power leg extension left
PPLER	Peak power leg extension right
PPLFR	Peak power leg flexion right
RFTL	Rectus femoris thickness on the left
RFTR	Rectus femoris thickness on the right
RFCSAL	Rectus femoris cross-sectional area on the left
RFCSAR	Rectus femoris cross-sectional area on the right
RFSL	Rectus femoris stiffness on the left
RFSR	Rectus femoris stiffness on the right
