# Supplementary material for: Effects of Blood Flow Restriction Resistance Exercise Versus Traditional Resistance Exercise in Voluntary Exhaustion on Quadriceps Muscle Adaptations in Untrained Young Males: A Randomized Trial
Source: Medicina (Kaunas). 2025 Apr 26;61(5):804. doi: 10.3390/medicina61050804 (PMC12113377; doi:10.3390/medicina61050804)
Supplement: Supplementary file 1 [file medicina-61-00804-s001.zip › medicina-3580285-supplementary.pdf]

CONSORT 2010 checklist of information to include when reporting a randomised trial*

Section/Topic	Item No	Checklist item	Reported on section
**Title and abstract**			
	1a	Identification as a randomised trial in the title	Title
	1b	Structured summary of trial design, methods, results, and conclusions (for specific guidance see CONSORT for abstracts)	Abstract
**Introduction**			
Background and objectives	2a	Scientific background and explanation of rationale	Introduction section/last paragraph
	2b	Specific objectives or hypotheses	Introduction section/last paragraph
**Methods**			
Trial design	3a	Description of trial design (such as parallel, factorial) including allocation ratio	Procedures subsection /First paragraph
	3b	Important changes to methods after trial commencement (such as eligibility criteria), with reasons	Participants subsection/First paragraph
Participants	4a	Eligibility criteria for participants	Participants subsection/First paragraph
	4b	Settings and locations where the data were collected	Methods /First subsection
Interventions	5	The interventions for each group with sufficient details to allow replication, including how and when they were actually administered	Procedures subsection
Outcomes	6a	Completely defined pre-specified primary and secondary outcome measures, including how and when they were assessed	Methods /First subsection
	6b	Any changes to trial outcomes after the trial commenced, with reasons	N/A

Sample size	7a	How sample size was determined	Participants subsection
	7b	When applicable, explanation of any interim analyses and stopping guidelines	Procedures subsection
Randomisation:			
Sequence generation	8a	Method used to generate the random allocation sequence	Procedures subsection
	8b	Type of randomisation; details of any restriction (such as blocking and block size)	Procedures subsection
Allocation concealment mechanism	9	Mechanism used to implement the random allocation sequence (such as sequentially numbered containers), describing any steps taken to conceal the sequence until interventions were assigned	Procedures subsection
Implementation	10	Who generated the random allocation sequence, who enrolled participants, and who assigned participants to interventions	Procedures subsection
Blinding	11a	If done, who was blinded after assignment to interventions (for example, participants, care providers, those assessing outcomes) and how	Methods /First subsection
	11b	If relevant, description of the similarity of interventions	N/A
Statistical methods	12a	Statistical methods used to compare groups for primary and secondary outcomes	Statistical analysis subsection
	12b	Methods for additional analyses, such as subgroup analyses and adjusted analyses	Statistical analysis subsection
**Results**			
Participant flow (a diagram is strongly recommended)	13a	For each group, the numbers of participants who were randomly assigned, received intended treatment, and were analysed for the primary outcome	Figure 1
	13b	For each group, losses and exclusions after randomisation, together with reasons	Figure 1
Recruitment	14a	Dates defining the periods of recruitment and follow-up	Figure 2
	14b	Why the trial ended or was stopped	N/A
Baseline data	15	A table showing baseline demographic and clinical characteristics for each group	Table 1
Numbers analysed	16	For each group, number of participants (denominator) included in each analysis and whether the analysis was by original assigned groups	Figure 1
Outcomes and	17a	For each primary and secondary outcome, results for each group, and the estimated effect size and its	Table 3

estimation		precision (such as 95% confidence interval)	
	17b	For binary outcomes, presentation of both absolute and relative effect sizes is recommended	N/A
Ancillary analyses	18	Results of any other analyses performed, including subgroup analyses and adjusted analyses, distinguishing pre-specified from exploratory	Results section
Harms	19	All important harms or unintended effects in each group (for specific guidance see CONSORT for harms)	Results section/first paragraph
**Discussion**			
Limitations	20	Trial limitations, addressing sources of potential bias, imprecision, and, if relevant, multiplicity of analyses	Discussion section/ last paragraph
Generalisability	21	Generalisability (external validity, applicability) of the trial findings	Discussion section
Interpretation	22	Interpretation consistent with results, balancing benefits and harms, and considering other relevant evidence	Discussion section
**Other information**			Methods /First subsection
Registration	23	Registration number and name of trial registry	
Protocol	24	Where the full trial protocol can be accessed, if available	Methods /First subsection
Funding	25	Sources of funding and other support (such as supply of drugs), role of funders	N/A

Citation: Schulz KF, Altman DG, Moher D, for the CONSORT Group. CONSORT 2010 Statement: updated guidelines for reporting parallel group randomised trials. BMC Medicine. 2010;8:18.
© 2010 Schulz et al. This is an Open Access article distributed under the terms of the Creative Commons Attribution License (http://creativecommons.org/licenses/by/2.0), which permits unrestricted use, distribution, and reproduction in any medium, provided the original work is properly cited.

*We strongly recommend reading this statement in conjunction with the CONSORT 2010 Explanation and Elaboration for important clarifications on all the items. If relevant, we also recommend reading CONSORT extensions for cluster randomised trials, non-inferiority and equivalence trials, non-pharmacological treatments, herbal interventions, and pragmatic trials. Additional extensions are forthcoming: for those and for up-to-date references relevant to this checklist, see www.consort-statement.org.
